# Antitumor properties of certain spirooxindoles towards hepatocellular carcinoma endowed with antioxidant activity

**DOI:** 10.1080/14756366.2020.1743281

**Published:** 2020-03-25

**Authors:** Sara T. Al-Rashood, Ahmed R. Hamed, Ghada S. Hassan, Hamad M. Alkahtani, Abdulrahman A. Almehizia, Amal Alharbi, Mohammad M. Al-Sanea, Wagdy M. Eldehna

**Affiliations:** a Department of Pharmaceutical Chemistry, College of Pharmacy, King Saud University, Riyadh, Saudi Arabia; b Department of Chemistry of Medicinal Plants, National Research Centre, Dokki, Egypt; c Biology Unit, Central Laboratory of the Pharmaceutical & Drug Industries Research Division, National Research Centre, Dokki, Egypt; d Department of Medicinal Chemistry, Faculty of Pharmacy, Mansoura University, Mansoura, Egypt; e Department of Pharmaceutical Chemistry, College of Pharmacy, Jouf University, Sakaka, Saudi Arabia; f Department of Pharmaceutical Chemistry, Faculty of Pharmacy, Kafrelsheikh University, Kafr El-Sheikh, Egypt

**Keywords:** Anti-proliferative, antioxidants, apoptosis, spiroxindoles, synthesis

## Abstract

In the current medical era, spirooxindole motif stands out as a privileged heterospirocyclic scaffold that represents the core for a wide range of bioactive naturally isolated products (such as Strychnofoline and spirotryprostatins A and B) and synthetic compounds. Interestingly, no much attention has been paid to develop spirooxindole derivatives with dual antioxidant and anticancer activities. In this context, a series of spirooxindoles 6a-p was examined for their anticancer effect towards HepG2 hepatocellular carcinoma and PC-3 prostate cancer cell lines. Spirooxindole 6a was found to be an efficient anti-proliferative agent towards both HepG2 and PC-3 cells (IC50 = 6.9 and 11.8 µM, respectively). Afterwards, spirooxindole 6a was assessed for its apoptosis induction potential in HepG2 cells, where its pro-apoptotic impact was approved via the significant elevation in the Bax/Bcl-2 ratio and the expression levels of caspase-3,

## Introduction

In the current medical era much attention has been paid to fight the extreme assembly of reactive oxygen species (ROS), such as hydrogen peroxide, hydroxyl radicals, or superoxide anion, that aggressively interact with natural macromolecules in different tissues by chain process resulting in an oxidative damage^1^^,^^2^. Biological systems have the capability to defend themselves against these ROS *via* enzymes (such as glutathione peroxidase, superoxide dismutase, and catalase) or by non-enzymatic mechanisms that involve organic antioxidants molecules (such as glutathione, vitamin C, vitamin E). These antioxidants are capable of direct scavenging the oxidant species through breaking of oxidation propagation chain reactions or through the generation of hydrogen atom in stoichiometric manner, hence preventing free radical-associated damage^1^^,^^2^.

Both depletion of antioxidant defences or abnormal ROS production, result in the oxidative stress state that implicated in the aetiology of a variety of disorders such as Alzheimer’s^3^, rheumatoid arthritis^4^, Huntington’s disease^5^, Parkinson’s disease^6^, coronary heart disease^7^, and insulin resistance^8^. Furthermore, elevated levels of ROS can promote tumorigenesis by initiation of mitochondrial or nuclear DNA mutations that promote neoplastic transformation, promoting genomic instability, or activating pro-oncogenic signalling pathways^9^. So, novel antioxidants featuring a variety of chemical structures are needed for prevention of cancer and to augment chemotherapies for different malignancies.

**Figure 1.**
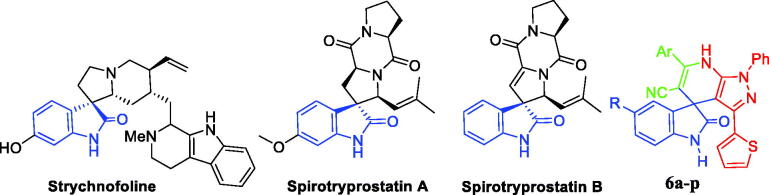
Chemical structure of some naturally isolated anticancer spirooxindoles, and the synthetic spirooxindoles **6a–p**.

Being abundant in nature, isatins have been found in fluids and tissues of mammals, in addition to natural products that are produced by a range of bacteria, plants, and invertebrates^10^. Isatin motif is a central privileged scaffold in a large number of bioactive natural and synthetic products that possess a spectrum of bioactivities such as antioxidant^11^ and anticancer activities^12–14^. On the other hand, the natural spirooxindole alkaloids were first isolated from plants of the *Apocynaceae* and *Rubiacae* families^15^. Thereafter, spirooxindole motif stands out as a privileged heterospirocyclic scaffold that represents the core for a wide range of naturally isolated products such as alstonisine^16^, horsfiline^17^, coerulescine^18^, and chitosenine^19^ with diverse bioactivity profiles. Strychnofoline, spirotryprostatin A and spirotryprostatin B (Figure 1) are other examples for natural spirooxindoles possessing anticancer activities^20^^,^^21^. Isolation and identification of such bioactive spirooxindole alkaloids have inspired the researchers and paved the way to design and synthesise various spirooxindole-based small molecules with promising biological activities such as antioxidant^22^ and anticancer^23–25^ activities.

Recently, we have reported a one-pot three-component synthesis of novel spirooxindole derivatives **6a–p** as efficient anti-proliferative agents towards the aggressive triple-negative breast cancer MDA-MB-231 cell line^25^. In the current study, the reported promising anti-proliferative impact of spirooxindoles **6a–p** spurred us to extend our investigations around their different potential biological activities, where they were evaluated for their potential antioxidant activity *via* assessing their DPPH radical scavenging capacities and Ferric reducing antioxidant power (FRAP). In addition, **6a–p** were examined for their anticancer effect towards HepG2 hepatocellular carcinoma and PC-3 prostate cancer cell lines. Furthermore, spirooxindole **6a** was then assessed for its apoptosis induction potential in HepG2 hepatocellular carcinoma cell line, so as to obtain some mechanistic insights for the anti-proliferative impact of the herein reported spirooxindoles. Finally, an *in silico* study was carried out by the use of the automated SwissADME tool to afford clues about the physicochemical properties, ADME parameters, drug-like nature and pharmacokinetic properties of spirooxindole **6a**.

## Materials and methods

### Chemistry

Spirooxindoles **6a–p** were prepared earlier by our group^25^.

### Biological evaluation

#### Anti-proliferative activity

##### Anti-proliferative activity towards HepG2 and PC-3 cell lines

Spirooxindoles **6a–p** were evaluated for their anti-proliferative potency towards HepG2 liver cancer and PC-3 prostate cancer cell lines. Both cells lines were cultured as monolayers in DMEM supplemented with 10% foetal bovine serum (FBS), L-glutamine (2 mM), penicillin (100 U/mL) and streptomycin sulphate (100 µg/mL). Then, cells were sub-cultured with trypsine/EDTA solution, counted with haemocytometer and plated onto 96-well plates (5000 cells/well) and left overnight so as to form a semi-confluent monolayer. A modified method was employed utilising the (3-[4,5-dimethylthiazol-2-yl] − 2,5-diphenyltetrazolium bromide (MTT) dye, as reported earlier^25–27^. IC_50_ values were calculated using non-linear regression curve fitting of the dose response plots on GraphPad Prism V.6.0 software. Assessment of morphological changes of HepG2 cells after treatment with the active compounds **6a**, **6e** and **6i** were carried out by phase contrast inverted microscope (Ziess, USA) and photomicrographs were taken using digital camera.

##### Apoptosis study

The levels of the apoptotic markers caspase-3, caspase-9, p53 and Bax, as well as the anti-apoptotic marker Bcl-2 were evaluated by the use of ELISA colorimetric kits per the manufacturer’s instructions, as reported earlier^28^^,^^29^. Furthermore, the pro-apoptotic potential of spirooxindole **6a** towards HepG2 cells was assessed using FITC Annexin V Apoptosis Detection Kit by flow cytometry, according to the manufacturer’s protocol and referring to the reported procedures^30^^,^^31^.

##### Cell cycle analysis

Cell cycle distributions in HepG2 cells were examined through PI staining and analysed by flow cytometry after treatment with spirooxindole **6a** at its IC_50_ (6.9 µM), as reported earlier^30^^,^^31^. The samples were analysed by a flow cytometer (BectonDickinson FACSCalibur, BD, USA) and data were analysed using the CellQuest software (Becton Dickinson).

#### Antioxidant activity

##### DPPH radical scavenging activity

The antioxidant potential of spirooxindoles **6a–p** against DPPH**˙** was evaluated as per previously described procedures by Brand-Williams et al.^32^. DPPH solution was prepared by dissolving 0.024 g DPPH in methanol (100 mL). In 96-well plates, × μL sample and 200-x μL DPPH solution were added. The plate was incubated for 30 min at room temperature in the dark, and finally, absorbance was recorded at 517 nm wavelength. Ascorbic acid was used as positive control. The percent radical scavenging potential was calculated for each compound *via* the following equation: Scavenging effect (%) = {(Absorbance of control − Absorbance of sample)/Absorbance of control} × 100.

##### Ferric reducing antioxidant power (FRAP)

The Benzie and Strain method^33^ was adopted to assess the ferric reducing antioxidant power (FRAP) for spirooxindoles **6a–p**. Absorbance of mixtures was recorded at 593 nm after incubation for 30 min at 37 °C. FRAP results were expressed as μM Fe^2+^ equivalents.

## Results and discussion

### Chemistry

**Scheme 1.**
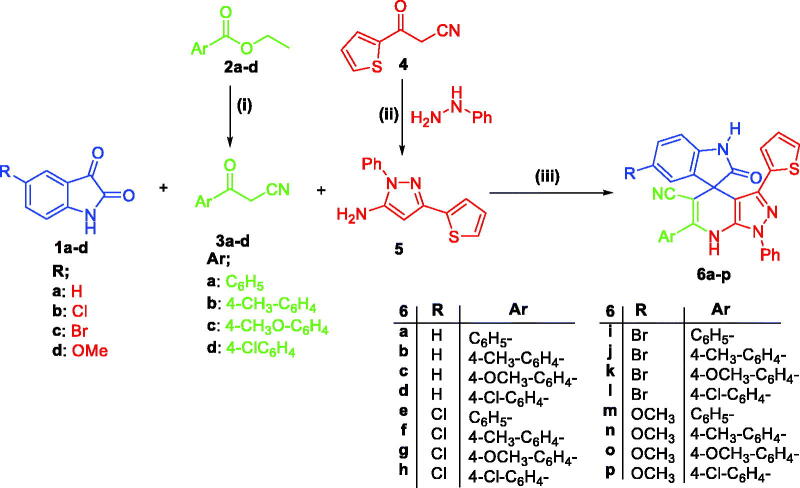
Synthesis of target compounds **6a–p**; *Reagents and conditions*: (**i**) CH_3_CN, DMF, NaH, benzene, reflux 4 h; (**ii**) Ethanol, phenylhydrazine, reflux 1 h; (**iii**) HOAc/H_2_O (1:1 v/v), heating at 120 °C, 8–11 h.

The synthetic pathway adopted for preparation of the desired spirooxindoles is illustrated in Scheme 1. First, ethyl benzoates **2a–d** were reacted with acetonitrile under reflux temperature in dry benzene and DMF, and in the presence of sodium hydride to furnish intermediates 3-oxo-3-phenylpropanenitriles **3a–d**. Furthermore, synthesis of 5-aminopyrazole **5** was achieved through the heterocyclocondensation of intermediate **4** with phenylhydrazine in ethyl alcohol under reflux temperature. Finally, the target spirooxindoles **6a–p** were prepared through a one-pot three-component reaction of equimolar amounts of isatins **1a–d**, 3-oxo-3-phenylpropanenitriles **3a–d**, and 1-phenyl-3-(thiophen-2-yl)-1*H*-pyrazol-5-amine **5** in HOAc/H_2_O mixture (1:1 v/v) at 70 °C (Scheme 1).

### Biological evaluation

#### Antitumor activity

##### Anti-proliferative activity towards HepG2 and PC-3 cell lines

Spirooxindoles **6a–p** were tested for their *in vitro* anti-HCC activity against HepG2 cell line, and for their anti-prostate cancer activity against PC-3 cell line by the use of MTT reduction assay^27^. The anticancer drug Adriamycin was co-assayed as the positive control. The anti-proliferative activities of tested spirooxindoles against the two cancer cell lines were measured as IC_50_ (in μM) value (the dose that affords a 50% inhibition of cell growth after 48 h of incubation) and displayed in Table 1.

**Table 1.** Anti-proliferative activities of spirooxindoles **6a–p** against HepG2 hepatocellular carcinoma and PC-3 prostate cancer cell lines.


Compound	R	Ar	IC_50_ (µM)	
			HepG2	PC-3
**6a**	H	C_6_H_5_––	6.9	11.8
**6b**	H	4–CH_3_–C_6_H_4_–	21.0	34.2
**6c**	H	4–OCH_3_–C_6_H_4_–	19.1	43.7
**6d**	H	4–Cl–C_6_H_4_–	49.1	>50.0
**6e**	Cl	C_6_H_5_–	8.4	13.5
**6f**	Cl	4–CH_3_–C_6_H_4_–	32.7	29.3
**6g**	Cl	4–OCH_3_–C_6_H_4_–	>50.0	>50.0
**6h**	Cl	4–Cl–C_6_H_4_–	19.9	>50.0
**6i**	Br	C_6_H_5_–	6.3	17.9
**6j**	Br	4–CH_3_–C_6_H_4_–	9.9	26.6
**6k**	Br	4–OCH_3_–C_6_H_4_–	13.2	39.1
**6l**	Br	4–Cl–C_6_H_4_–	12.7	>50.0
**6m**	OCH_3_	C_6_H_5_–	30.6	24.3
**6n**	OCH_3_	4–CH_3_–C_6_H_4_–	>50.0	42.8
**6o**	OCH_3_	4–OCH_3_–C_6_H_4_–	>50.0	>50.0
**6p**	OCH_3_	4–Cl–C_6_H_4_–	>50.0	>50.0
**Adriamycin**			0.12	0.62

Investigation of the obtained results in Table 1 revealed that the tested spirooxindoles were more effective towards HCC HepG2 cells rather than prostate cancer PC-3 cells, except compounds **6f**, **6 m** and **6n** which caused slightly enhanced growth inhibitory activity against PC-3 (IC_50_ = 29.3, 24.3 and 42.8 µM, respectively) than HepG2 cells (IC_50_ = 32.7, 30.6 and >50.0 µM, respectively).

**Figure 2.**
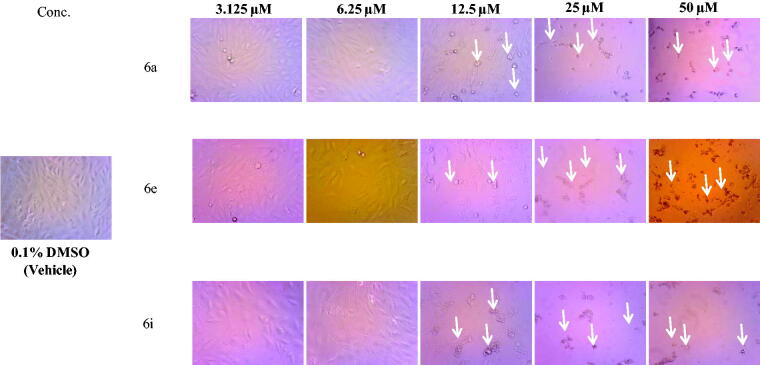
Morphological changes following 48 h exposure of HepG2 cells to indicated concentrations of **6a**, **6e** and **6i**. Signs of toxicity indicated with arrows represent cell rounding, shrinkage and/or loss of monolayer integrity. Total magnification = 300.

Regarding the activity towards HepG2 cells, the data displayed in Table 1 ascribed to the tested spirooxindoles excellent to weak efficacy in inhibiting the growth of the HCC HepG2 cells (IC_50_ values ranging between 6.3 and 49.1 µM, Table 1), except compounds **6 g** and **6n–6p** which failed to inhibit the growth of HepG2 cells up to 50 μM. In particular, spirooxindoles **6a** and **6i** were found to be the most potent derivatives in this study against HepG2 cells with IC_50_ values equal to 6.9 and 6.3 μM, respectively. In addition, spirooxindoles **6e** and **6j** exhibited good activity towards HepG2 cells (IC_50_ = 8.4 and 9.9 µM, respectively), whereas, compounds **6 b**, **6c**, **6 h**, **6k** and **6 l** elicited moderate potency against HepG2 cells with IC_50_ range: 12.7 − 21.0 µM. Morphological assessment revealed the toxic effects of spirooxinodoles **6a**, **6e** and **6i** as presented in Figure 2 including dramatic cellular effects including cell rounding, shrinkage and monolayer disruption.

On the other hand, it was apparent from the obtained results that most of the prepared spirooxindoles possessed moderate to modest growth inhibitory activity towards prostate cancer PC-3 cells with IC_50_ in the range of 11.8 − 43.7 µM. Compounds **6a** and **6e** were found to be the most potent spirooxindoles towards PC-3 cells with IC_50_ values equal to 11.8 and 13.5 μM, respectively. Moreover, spirooxindoles **6i, 6j** and **6 m** were moderately active against PC-3 cells with (IC_50_ = 17.9, 26.6 and 24.3 µM, respectively).

Observing the aforementioned results for the anti-proliferative activity MTT assay against HepG2 cells, certain structure activity relationships could be concluded. Firstly, we examined the effect of C-5 substitution of the indoline moiety. 5-Br substituted spirooxindoles **6i–6l** displayed more enhanced anti-proliferative activity (IC_50_ = 6.3, 9.9, 13.2 and 12.7 µM, respectively) than their corresponding unsubstituted spirooxindoles **6a–6d** (IC_50_ = 6.9, 21.0, 19.1 and 49.1 µM, respectively), 5-Cl substituted spirooxindoles **6e–6h** (IC_50_ = 8.4, 32.7, >50.0 and 19.9 µM, respectively), and 5-OCH_3_ substituted spirooxindoles **6 m–6p** (IC_50_ = 30.6, >50.0, >50.0 and >50.0 µM, respectively), which highlighted that C-5 substitution of the indoline moiety with the large lipophilic Br group is more advantageous for the growth inhibitory activity against HepG2 cells than substitution with smaller lipophilic (as Cl and OCH_3_) groups and unsubstitution.

We then explored the impact of substitution of the pendant phenyl group at C-6 of pyrazolo[3,4-*b*]pyridine moiety on the activity against HepG2 cells. Spirooxindoles **6a**, **6e**, **6i** and **6 m** that were entailed with an unsubstituted phenyl group showed better activity (IC_50_ = 6.9, 8.4, 6.3 and 30.6 µM, respectively) than their corresponding 4-substitutedphenyl counterparts **6c–d** (IC_50_ range: 213 − 49.1 µM), **6f–h** (IC_50_ range: 19.9 – > 50 µM), **6j–l** (IC_50_ range: 9.9 − 13.2 µM) and **6n–p** (IC_50_ > 50 µM), suggesting that incorporation of an unsubstituted phenyl group at C-6 of pyrazolo[3,4-*b*]pyridine moiety is indispensable for the anti-proliferative activity towards HCC HepG2 cells.

On the other hand, spirooxindoles **6a–c**, **6e**, **6f**, **6i**-**k** and **6 m**, with dual activity against both here examined HepG2 and PC-3 cancer cell lines, were tested for their potential cytotoxic impact towards the non-tumorigenic human breast MCF-10A cell line in order to explore the safety profile of the target spirooxindole scaffold towards the normal cells, Table 2.

**Table 2.** Cytotoxic action of spirooxindoles **6a–c**, **6e**, **6f**, **6i**–**k** and **6 m** towards non-tumorigenic human MCF-10A cell line, and selectivity index (*S. I*.) for tumour cells (MCF-10A/HepG2).

Compound	IC_50_ (µM)		Tumour *S. I*.
	MCF-10A	HepG2	
**6a**	94.56	6.9	13.7
**6b**	121.61	21.0	5.8
**6c**	141.30	19.1	7.4
**6e**	69.74	8.4	8.3
**6f**	158.79	32.7	4.9
**6i**	76.94	6.3	12.2
**6j**	146.41	9.9	14.8
**6k**	136.27	13.2	10.3
**6m**	185.96	30.6	6.1

The tested spirooxindoles **6a–c**, **6e**, **6f**, **6i**-**k** and **6 m** exerted non-significant cytotoxicity towards the non-tumorigenic MCF-10A cells with IC_50_ values spanning between 69.74 and 185.96 µM, respectively. In addition, the calculated for the tested spirooxindoles *S. I*. lied in the range: 4.9–14.8. Superiorly, spirooxindoles **6a** and **6j** displayed excellent safety profile with *S. I*. equal 13.7 and 14.8, respectively, Table 2.

##### Flow cytometric analysis and apoptotic studies in HepG2 cells

Spirooxindole **6a** was found to be an efficient anti-proliferative agent towards both HepG2 and PC-3 cells (IC_50_ = 6.9 and 11.8 µM, respectively, Table 1), in addition to its promising antioxidant activities (Tables 5 and 6). Accordingly, spirooxindole **6a** was selected for further evaluations to acquire mechanistic insights into its anti-proliferative activity against HepG2 cells.

##### Cell cycle analysis

**Figure 3.**
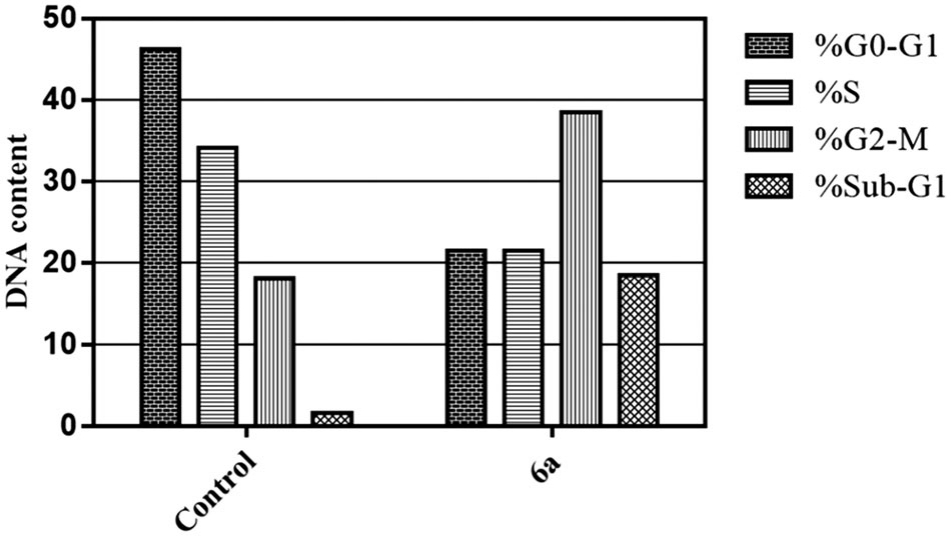
Effect of spirooxindole **6a** on the phases of cell cycle of HepG2 cells.

The impact of spirooxindole **6a** on the normal cell cycle progression was investigated using flow cytometric analysis of the DNA ploidy in HepG2 cells. Exposure of HepG2 cells to spirooxindole **6a** at its IC_50_ (6.9 µM) for 24 h revealed the ability of **6a** to disrupt the normal cell cycle by decreasing both G0-G1 and S phases by approximately 0.53- and 0.37-folds with respect to the control (Figure 3). Furthermore, both Pre-G and G2/M phases of the treated HepG2 cell were significantly increased by 11.9- and 2.1-folds related to the control (Figure 3). Alteration of the Pre-G phase and arrest of G2-M phase may imply apoptosis as a potential mechanism for **6a**-induced cancer cell death.

##### Effect of 6a on the levels of the apoptotic markers (Bax, caspase-3, caspase-9 and p53), and the anti-apoptotic marker Bcl-2

To investigate the ability of spirooxindole **6a** to provoke apoptosis, herein we assessed the expression levels of the hallmark parameters of apoptosis in HepG2 cells upon treatment with **6a** at its IC_50_ (6.9 µM) (Tables 3 and 4). In this study, exposure of HepG2 cells to spirooxindole **6a** resulted in a significant 5.7-fold increase in the expression of the pro-apoptotic protein Bax, with concurrent significant down-regulation of the expression levels of the anti-apoptotic protein Bcl-2 by ∼ 42% compared to the control (Table 3). Analysing these results declared that spirooxindole **6a** boosted the Bax/Bcl-2 ratio nine folds in comparison to the control. The Bax/Bcl-2 ratio is considered as a decisive value that gives a real insight to the overall pro-apoptotic impact of the examined compound.

**Table 3.** Effect of compound **6a** on the expression levels of Bax and Bcl-2 in HepG2 cells treated with the compound at its IC_50_.

Comp.	Bax ng/mL	Bcl-2 ng/mL	Bax/Bcl-2 ratio
**Control**	40.63	4.34	9.4
**6a**	232.2 (5.7)*	2.51 (0.57)*	92.5

*Numbers given between parentheses are the numbers of folds of control.

**Table 4.** Effect of compound **6a** on the expression levels of active caspases-3 and -9, and p53 in HepG2 cells treated with the compound at its IC_50_.

Comp.	Caspase-3 pg/mL	Caspase-9 ng/mL	p53 pg/mL
**Control**	53.57	2.19	77.15
**6a**	475.9 (8.88)*	26.92 (12.27)*	774.5 (10.03)*

*Numbers given between parentheses are the numbers of folds of control.

The elevated Bax/Bcl-2 ratio aroused the assessment of the expression levels of the pro-apoptotic active caspase-3, caspase-9 and p53 tumour suppressor protein. Treatment of HepG2 cells with spirooxindole **6a** resulted in a significant elevation in the expression levels of caspase-3, caspase-9 and p53 proteins by about 8.88, 12.27 and 10.03 folds, respectively, compared to control (Table 4).

The simultaneous up-regulation of the downstream caspase 3, the hallmark key player and the key executor of apoptosis, alongside to p53 highlighted that a cascade of apoptotic markers was activated as a consequence of Bax/Bcl2 elevation that eventually resulted in apoptosis.

##### Annexin V-FITC apoptosis assay

**Figure 4.**
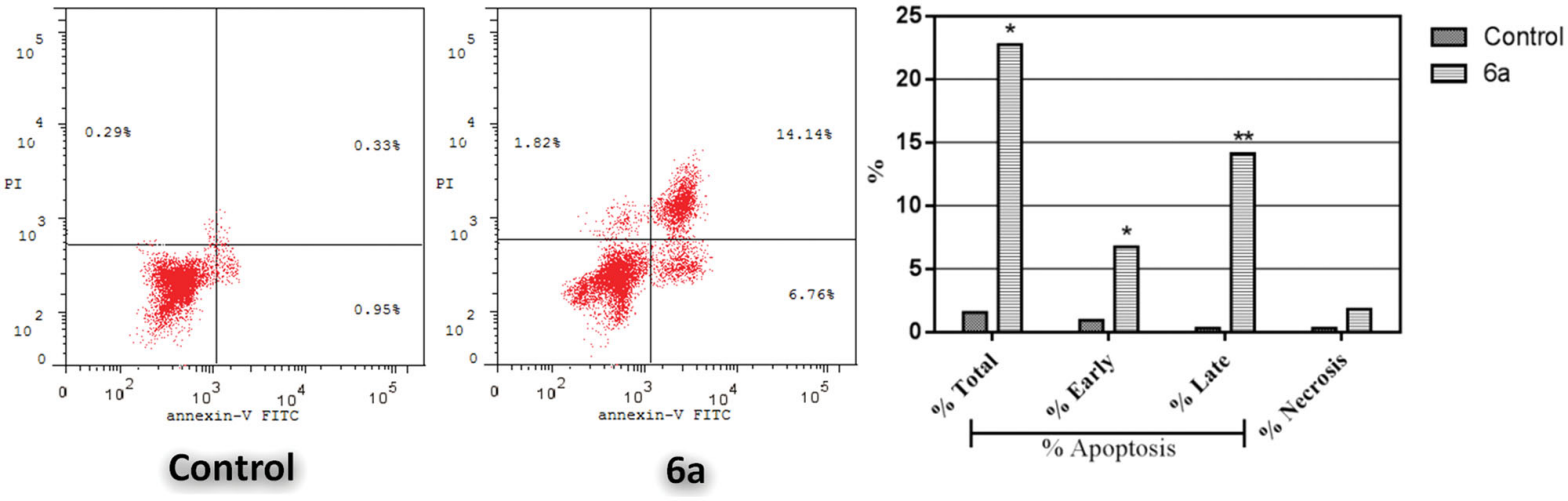
Effect of spirooxindole **6a** on the percentage of annexin V-FITC-positive staining in HepG2 cells. The experiments were done in triplicates. The four quadrants identified as: LL: viable; LR: early apoptotic; UR: late apoptotic; UL: necrotic.

To confirm the apoptotic effect of spirooxindole **6a** in HepG2 cells, Annexin-V FTIC/PI dual staining assay was carried out at IC_50_ of **6a** (6.9 µM). Examining the results of this flow cytometric analysis (Figure 4), revealed that HepG2 cells treated with spirooxindole **6a** exhibited a significant increase in the percentage of annexin-V positive cells indicating an early (lower right quadrant) and late (upper right quadrant) apoptosis from 0.95% to 6.76%, and from 0.33% to 14.14%, respectively, which comprises about 16.3-folds total increase with respect to control (Figure 4).

#### Antioxidant activity

##### DPPH radical scavenging activity

The DPPH (2,2-diphenyl-1-picrylhydrazyl) radical scavenging activity assay is considered as one of the most common and simple methods for measuring the antioxidant ability to trap free radicals. DPPH is a commercially available stable free radical that possesses the ability to accept an electron to produce a stable molecule. Substances that have the potential to act as electron donors result in reduction of this DPPH radical to the corresponding DPPH_2_ disclosing their antioxidant potential. The principle of the method relies upon measuring the absorbance of the odd electrons of DPPH at 517 nm. The absorbance decreases, in the presence of free radical scavengers, proportional to the decrease of the DPPH radical concentration^34^. In the current study, the scavenging effect of compounds **6a–p** was evaluated *via* the DPPH radical scavenging assay, and compared with the standard antioxidant; ascorbic acid. The results, summarised in Table 5, are expressed in terms of IC_50_ values that produce 50% inhibition of the DPPH radical; where the lower is this value, the more active is the examined compound.

From the obtained results, it was obvious that most of the prepared spirooxindoles **6a–p** exhibited excellent to moderate DPPH radical scavenging capacities with IC_50_ spanning in the range: 28.07 − 113.45 µg/mL. Superiorly, both spirooxindoles **6a** and **6 m** emerged as the most efficient DPPH radical scavengers with IC_50_ values of 29.76 ± 1.82 and 28.07 ± 1.63 µg/mL, respectively, which are 1.6-fold more potent than the standard antioxidant Ascorbic acid (IC_50_ = 45.89 ± 2.39 µg/mL). Also, compounds **6c–6f**, **6i**, **6j** and **6 l** showed higher DPPH radical scavenging capacities (IC_50_ range: 33.43 ± 1.24 − 43.42 ± 3.04 µg/mL) than Ascorbic acid. Furthermore, spirooxindoles **6 h**, **6k** and **6n–6p** displayed moderate antiradical activity with IC_50_ values of 46.29 ± 2.18, 69.25 ± 4.25, 46.29 ± 2.52, 58.82 ± 3.14 and 55.05 ± 3.41 µg/mL, respectively. Contrariwise, compounds **6 b** and **6 g** elicited weak DPPH˙ scavenging activity with IC_50_ values of 113.45 ± 5.59 and 97.47 ± 5.62 µg/mL, respectively.

##### Ferric reducing antioxidant power (FRAP)

FRAP assay is a widely used antioxidant assay that uses antioxidant substances as reducing agents in a redox-linked colorimetric reaction, wherein Fe^3+^ is reduced to Fe^2+^ at acidic pH that results in a formation of a coloured ferrous-probe complex from a colourless ferric-probe complex^35^. In this assay, the electron donation capacity (reflecting the electron transfer ability) of the examined compounds is evaluated.

In this study, all spirooxindoles **6a–p** herein reported were evaluated for their potential FRAP. The results were expressed as µM Fe^2+^ equivalents and presented in Table 6, where the higher µM Fe^2+^ equivalents value means higher FRAP and subsequently higher antioxidant capacity. As in the above DPPH assay, both spirooxindoles **6a** and **6 m** exhibited the best FRAP activity (11.98 and 12.13 µM, respectively) that is comparable to the activity of the reference Ascorbic acid (13.42 µM). Moreover, compounds **6c–6f**, **6 h–6j**, **6 l** and **6n–6p** possessed good reducing capacity in this assay with FRAP range: 10.12 − 11.90 µM.

**Table 5.** The *in vitro* antioxidant activity of spirooxindoles (**6a–p**) in DPPH**˙** scavenging assay.


Compound	R	Ar	IC_50_ (µg/mL)^a^
**6a**	H	C_6_H_5_–	29.76 ± 1.82
**6b**	H	4–CH_3_–C_6_H_4_–	113.45 ± 5.59
**6c**	H	4–OCH_3_–C_6_H_4_–	43.04 ± 2.39
**6d**	H	4–Cl–C_6_H_4_–	33.43 ± 1.24
**6e**	Cl	C_6_H_5_–	36.03 ± 1.37
**6f**	Cl	4–CH_3_–C_6_H_4_–	40.31 ± 2.61
**6g**	Cl	4–OCH_3_–C_6_H_4_–	97.47 ± 5.62
**6h**	Cl	4–Cl–C_6_H_4_–	46.29 ± 2.18
**6i**	Br	C_6_H_5_–	36.38 ± 2.71
**6j**	Br	4–CH_3_–C_6_H_4_–	43.42 ± 3.04
**6k**	Br	4–OCH_3_–C_6_H_4_–	69.25 ± 4.25
**6l**	Br	4–Cl–C_6_H_4_–	41.36 ± 2.17
**6m**	OCH_3_	C_6_H_5_–	28.07 ± 1.63
**6n**	OCH_3_	4–CH_3_–C_6_H_4_–	46.29 ± 2.52
**6o**	OCH_3_	4–OCH_3_–C_6_H_4_–	58.82 ± 3.14
**6p**	OCH_3_	4–Cl–C_6_H_4_–	55.05 ± 3.41
**Ascorbic acid**	–	–	45.89 ± 2.39

**^a^**IC_50_ values are the mean ± SD of three separate experiments.

**Table 6.** The *in vitro* antioxidant activity of spirooxindoles (**6a–p**) in FRAP assay.

Compound	µM (Fe^2+^ Eq.)
**6a**	**11.98**
**6b**	6.933
**6c**	10.32
**6d**	10.99
**6e**	10.12
**6f**	11.36
**6g**	7.79
**6h**	11.35
**6i**	11.88
**6j**	10.34
**6k**	8.499
**6l**	11.59
**6m**	**12.13**
**6n**	11.58
**6o**	11.90
**6p**	11.18
**Ascorbic acid**	13.42

### Physicochemical, ADME and pharmacokinetic properties prediction

For prediction of the pharmacokinetic properties, ADME parameters and drug-like nature of spirooxindole **6a**, the automated *SwissADME* online web tool^36^ was employed. Articulating such properties is of great significance in the context of pharmaceutical chemistry to develop efficient clinical candidates.

**Figure 5.**
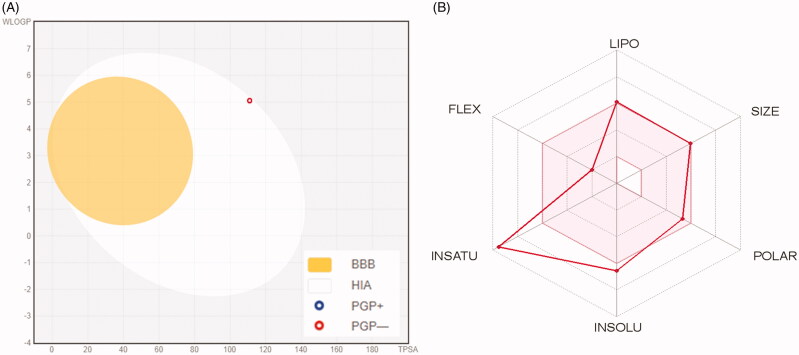
(**A**) Predicted Boiled-Egg plot from *swissADME* online web tool for spirooxindole **6a**; (**B**) Bioavailability radar chart for spirooxindole **6a**; The pink area represents the range of the optimal property values for oral bioavailability and the red line is spirooxindole **6a** predicted properties.

Figure 5(A) displays the BOILED-Egg graph of the WLOGP vs. TPSA (topological polar surface area) for spirooxindole **6a**^37^, where it is located in the region of human intestinal absorption (HIA) with no BBB permeability so it is anticipated to display low occurrence for CNS side effects. Also, this graph revealed that spirooxindole **6a** is not a P-glycoprotein substrate (PGP−), therefore it is not susceptible to the efflux mechanism carried out by this transporter that utilised by many cancer cell lines as a drug-resistance mechanism^38^^,^^39^. Figure 5(B) represents the bioavailability radar chart for spirooxindole **6a**, which consists of six axes for six key properties for oral bioavailability; polarity (POLAR), solubility (INSOLU), lipophilicity (LIPO), flexibility (FLEX), saturation (INSATU) and size (SIZE) ^39^^,^^40^. The range for optimal property values is displayed as a pink area, whereas the red line represents the predicted properties for the examined molecule.

Furthermore, as shown in Table 7, spirooxindole **6a** showed high predicted level of gastrointestinal absorption, and predicted to be inhibitor of three of CYP isoforms (CYP1A2, CYP2C9 and CYP2C19) whereas predicted to be non-inhibitor of CYP3A4 and CYP2D6 isoforms.

**Table 7.** *In silico* predictions of the pharmacokinetics properties for spirooxindole **6a**.

Cpd.	GIA	BBB	P-gp substrate	CYP1A2 inhibitor	CYP2C9 inhibitor	CYP2C19 inhibitor	CYP3A4 inhibitor	CYP2D6 inhibitor
**6a**	High	No	No	Yes	Yes	Yes	No	No

**GIA:** gastrointestinal absorption; **BBB:** blood-brain barrier permeation; **P-gp:** permeability glycoprotein**; CYP1A2, CYP2C9, CYP2C19, CYP3A4** and **CYP2D6** are the five major isoforms of cytochromes P450.

Moreover, *SwissADME* tool showed that spirooxindole **6a** fulfils the drug-likeness characteristics as defined by the major pharmaceutical companies (Table 8) and passed their filters; Lipinski’s (Pfizer)^41^, Veber’s (GSK)^42^ and Egan’s (Pharmacia)^43^ filters, whereas it failed to pass Ghose’s (Amgen) filter due to two violations; molecular weight > 480, and molar refractivity >130^44^. However, it was not classified as lead-like because its molecular weight is higher than 350 and its predicted XLOGP3^45^ exceeded 3.5.

**Table 8.** *In silico* predictions of the drug-likeness properties for spirooxindole **6a**.

Cpd.	Lipinski #violations	Ghose #violations	Veber #violations	Egan #violations	PAINS #alerts	Brenk #alerts	Bioavailability Score
**6a**	0	2	0	0	0	0	0.55

All calculations were performed using SwissADME [36].

In summary, this *in silico* study for predicting the physicochemical and pharmacokinetic properties of spirooxindole **6a** revealed that it is not only with promising antioxidant and anti-proliferative biological activities, but also with promising pharmacokinetic properties.

## Conclusions

In summary, a series of spirooxindole-based derivatives (**6a–p**) was examined for its anticancer effect towards HepG2 hepatocellular carcinoma and PC-3 prostate cancer cell lines. Spirooxindoles **6a–p** were more effective towards HCC HepG2 cells rather than prostate cancer PC-3 cells, except compounds **6f**, **6 m** and **6n.** In particular, spirooxindoles **6a** and **6i** were found to be the most potent derivatives in this study against HepG2 cells with IC_50_ values equal to 6.9 and 6.3 μM, respectively. The SAR outcomes highlighted that C-5 substitution of the indoline moiety with a large lipophilic Br group, and incorporation of unsubstituted phenyl group at C-6 of pyrazolo[3,4-b]pyridine moiety are indispensable factors for the growth inhibitory activity against HepG2 cells. Furthermore, spirooxindole **6a** was assessed for its apoptosis induction potential in HepG2 cells, where its pro-apoptotic impact was approved via the significant elevation in the Bax/Bcl-2 ratio and the expression levels of caspase-3, caspase-9 and p53 proteins by about 9, 8.8, 12.2 and 10 folds, respectively, and via the significant increase in the annexin V-FITC positive apoptotic cells percent. Besides, **6a–p** were screened for their potential antioxidant activity *via* assessing their DPPH radical scavenging capacities and Ferric reducing antioxidant power (FRAP). The obtained results indicated that spirooxindoles **6a** and **6 m** are the most efficient DPPH radical scavengers (IC_50_ = 29.76 ± 1.82 and 28.07 ± 1.63 µg/mL, respectively), and exhibited the best FRAP activity (11.98 and 12.13 µM, respectively). Finally, an *in silico* study was carried out to predict the physicochemical and pharmacokinetic properties of spirooxindole **6a**, which revealed that 6a is not only with promising antioxidant and anti-proliferative activities, but also with proper drug-likeness and pharmacokinetic properties.
